# Gynecological Surgery and COVID-19: What is the Impact and How Should I Manage it?*

**DOI:** 10.1055/s-0040-1715146

**Published:** 2020-07

**Authors:** Julio Cesar Rosa-e-Silva, Paulo Ayroza Ribeiro, Luiz Gustavo Oliveira Brito, Mariano Tamura Vieira Gomes, Sergio Podgaec, Helizabet Salomão Abdalla Ayroza Ribeiro, Carlos Augusto Pires Costa Lino, Ricardo Quintairos, Walquiria Quida Salles Pereira Primo, Agnaldo Lopes da Silva Filho

**Affiliations:** 1 Faculdade de Medicina de Ribeirão Preto, Universidade de São Paulo, Ribeirão Preto, SP, Brazil; 2 Faculdade de Ciências Médicas da Santa Casa de São Paulo, São Paulo, SP, Brazil; 3 Departamento de Tocoginecologia, Faculdade de Ciências Médicas, Universidade Estadual de Campinas, Campinas, SP, Brazil; 4 Escola Paulista de Medicina, Universidade Federal de São Paulo, São Paulo, SP, Brazil; 5 Hospital Israelita Albert Einstein, São Paulo, SP, Brazil; 6 Faculdade de Medicina, Universidade de São Paulo, São Paulo SP, Brazil; 7 Instituto de Perinatologia da Bahia, Salvador, BA, Brazil; 8 Hospital Porto Dias, Belém, PA, Brazil; 9 Faculdade de Medicina, Universidade de Brasilia, Brasília, DF, Brazil; 10 Universidade Federal de Minas Gerais, Belo Horizonte, MG, Brazil

**Keywords:** COVID-19, laparoscopy, hysteroscopy, gynecological surgery, COVID-19, Laparoscopia, Histeroscopia, Cirurgia ginecológica

## Abstract

It is estimated that around 28 million surgeries will be postponed or canceled worldwide as a result of this pandemic, causing a delay in the diagnosis and treatment of more than 2 million cancer cases. In Brazil, both the National Health Agency (ANS) and National Health Surveillance Agency (ANVISA) advised the postponement of elective and non-essential surgeries, causing a considerable impact on the number of surgical procedures that decreased by 33.4% in this period. However, some women need treatment for various gynecological diseases that cannot be postponed. The purpose of this article is to present recommendations on surgical treatment during the COVID-19 pandemic.

## Resumo

Estima-se que cerca de 28 milhões de cirurgias sejam postergadas ou canceladas no mundo em decorrência desta pandemia, causando atraso no diagnóstico e tratamento de mais de 2 milhões de casos oncológicos. No Brasil, tanto a ANS (Agencia Nacional de Saúde) como a ANVISA (Agencia Nacional de Vigilância Sanitária) orientaram o adiamento das cirurgias eletivas e não essenciais, tendo um impacto considerável no número de procedimentos cirúrgicos com diminuição de 33,4% neste período no Brasil. No entanto, algumas mulheres necessitam de tratamento para várias doenças ginecológicas, algumas das quais não podem ser adiadas. O objetivo deste artigo é apresentar recomendações sobre o tratamento cirúrgico durante a pandemia de COVID-19.

## Introduction

The first human coronaviruses were isolated for the first time in 1937. In 1965, the virus was described as coronavirus because of its profile under microscopy that resembles a crown. Coronavirus is a family of viruses that cause respiratory infections.

Since the beginning of February, the World Health Organization (WHO) has officially called the disease caused by the new coronavirus as Covid-19. COVID stands for COrona VIrus Disease, while ‘19’ refers to 2019, when the first cases in Wuhan, China, were publicly released by the Chinese government in late December.

The disease pandemic (COVID-19) is a global health emergency and governments around the world are trying to curb the rate of infection by using complete or partial population isolation for the reduction of people's mobility, thereby slowing the spread of the disease. At this moment, Brazil is one of the countries most affected by the virus. The plan is that different phases of the lockdown will contain the circulation of the virus and prevent the complete socioeconomic collapse. The extent of population isolation depends on the situation of the epidemic, with three different phases: (1) total lockdown in the emergency period during the peak of the epidemic; (2) an intermediate period started when the contagion curve begins to fall; and (3) progressive resumption of all normal activities. In Brazil, we are starting to resume activities in some regions, while others are still applying total social isolation.

In many cases, COVID-19 pneumonia requires hospitalization and intensive treatment. Currently, more than 7 million people have already been diagnosed with the disease in the world and about 400 thousand people have lost their lives as a result, with a lethality rate of more than 5%. In Brazil, more than 700 thousand people were diagnosed with the disease and about 37 thousand people died. As the surgery is a high-risk situation for the transmission of respiratory infections,^1^ any type of elective surgical treatment should be evaluated and postponed, if possible.

It is estimated that around 28 million surgeries are postponed or canceled worldwide due to this pandemic, causing a delay in the diagnosis and treatment of more than 2 million cancer cases. In Brazil, both the National Health Agency (Portuguese acronym: ANS) and the National Health Surveillance Agency (Portuguese acronym: ANVISA) advised the postponement of elective and non-essential surgeries. This had a considerable impact on the number of surgical procedures, which decreased by 33.4% in the country in this period.

However, some women need treatment for various gynecological diseases that cannot be postponed. Conservative non-surgical treatment, including pharmacological and alternative therapies, can be implemented even if there is no high-level scientific evidence recommending it. Performing surgery on potential patients with COVID-19 poses a high-risk challenge and several international societies have recommended a non-surgical approach when possible.

In China, a group of asymptomatic patients infected with SARS-COV-2 who underwent a surgical procedure had an unfavorable evolution; 100% of patients developed symptoms, 44.1% required intensive care and 20.5% died.^2^

Guidelines on the surgical treatment of gynecological diseases at this time are scarce and mostly based on expert opinion, current information available and recommendations from international scientific societies. The purpose of this article is to present recommendations on surgical treatment during the COVID-19 pandemic.

## SARS-CoV-2 and COVID-19: Disease and Transmission

The virus that causes COVID-19 is transmitted by saliva and respiratory droplets. Airborne transmission is possible because the virus remains in the air for long periods of time and can be transmitted to others at distances up to 1 meter,^3^ as well as in specific circumstances that generate aerosols, such as endotracheal intubation, airway manipulation and probably surgery.

The SARS-CoV-2 is present in several cavities of the body of infected patients therefore, during surgery, it can be disseminated through the spray generated by surgical instruments, because the aerosol may contain the virus or parts of it. Some authors suggest the virus remains viable in the aerosol for at least three hours.^4^

However, there is no evidence available from the current pandemic or from previous epidemics to conclude that respiratory viruses can be transmitted from patients to health professionals in the operating room via the abdominal route.^5^

The European Society of Gynecological Endoscopy (ESGE)^6^ and the American Association of Gynecological Laparoscopists (AAGL)^7^ have issued recommendations to continue performing minimally invasive surgeries with the adoption of specific care measures, such as the reduction of surgery time; dioxide carbon (CO2) leakage through trocars; aerosol production; and the spread of droplets of blood or fluid. For these purposes, it is useful to employ a plume filtration device coupled to the trocars and avoid rapid deflation or loss of pneumoperitoneum when changing instruments or extracting surgical parts.

All surgical procedures must be considered high risk, because asymptomatic patients may be carrying the virus. Even in vaginal or laparotomy approaches with the use of electrosurgical instruments, there may be formation of aerosol containing viral particles. Thus the importance of implementing measures to minimize these risks, such as performing dissection and vascular control using non-electrosurgical techniques whenever possible; using electrosurgical and ultrasonic devices when needed, in order to minimize aerosol production and avoid long drying times; using suction devices; and minimizing the spread of droplets of blood or fluids.

However, in choosing the surgical route, the presence of some patient comorbidity that results in greater morbidity due to laparotomic procedures, longer hospitalization period and higher risk of nosocomial infection should be considered.

Careful anamnesis with search for signs or symptoms of the disease (fever, dry cough, fatigue, shortness of breath, muscle pain, sore throat, diarrhea, nausea or vomiting and runny nose) and a history of recent contact with people with COVID-19 or flu-like symptoms, as well as detailed physical examination seem to be effective in detecting patients at risk.

Testing for SARS-CoV-2 (RT-PCR) or serological tests before elective surgery are essential to protect patients and healthcare professionals.^8^ The type of screening will depend on availability of the health system and the patient's signs and symptoms. However, the false-negative rate of exams and the appropriate period for their performance must always be taken into consideration. If patients cannot be tested prior to surgery, they must be considered potentially positive and all care with the mobilization of equipment and adequate protection for health professionals must be employed.

Currently, several types of tests can be used for virus detection or to check the individual has already had contact with it.

- RT-PCR: considered the gold standard in the diagnosis of COVID-19. Confirmation through the detection of SARS-CoV-2 RNA in the analyzed sample, preferably obtained from nasopharyngeal scraping (increased sensitivity 10-15% in relation to the oropharynx) performed between the third and tenth day of symptom onset.
- Serological tests: serology, unlike RT-PCR, checks the body's immune response to the virus. This is done by detecting IgM and IgG antibodies in people who have been exposed to the SARS-CoV-2. In this case, the test is performed on a patient's blood sample. Some laboratories offer the dosage of IgA, but its sensitivity is lower than that of IgG and IgM.
- Rapid tests: two types of rapid tests are available: antigen tests (detect proteins from the virus at the stage of infection activity) and antibodies tests (identify an immune response of the body in relation to the virus). The advantage of these tests would be their rapid results, although the existing ones have very low sensitivity and specificity in comparison to other methodologies.

Many laboratories can take up to 48 hours to perform the exams, and this fact must always be taken into account (Table 1).

**Table 1:** Sensitivity of tests for COVID 19

	Days after the onset of symptoms		
Test for SARS- CoV -2	1–7	8–14	15–39
RT-PCR	67%	54%	45%
Total antibodies	38%	90%	100%
IgM	29%	73%	94%
IgG	19%	54%	80%

**Source:** Adapted from Zhao et al.^9^

## How Sensitive are these Tests?

### When to use these Tests?

We will divide patients into asymptomatic and symptomatic for a better understanding of all and to help establish the procedure with a patient who needs elective, urgent or emergency surgery throughout this period:

- **Asymptomatic patient:** asymptomatic patient, afebrile, without a history of fever, without current respiratory symptoms or in the last 14 days. The patient will not always report these complaints actively and the professional assisting her should seek the information. This patient could be released for hospitalization and should undergo RT-PCR testing before the surgical procedure as a guarantee for her and the health team assisting her. Remember the sensitivity limitations of this test in asymptomatic patients. If the test is positive, the decision made in agreement with the patient may be to proceed with the surgery and assume greater risks of serious complications in the postoperative period, or postpone the procedure to a safer time, if possible. If RT-PCR is negative, the serological test can be performed. If IgM and IgG are negative, the patient probably has not yet had contact with the virus and could have her surgical procedure performed with the necessary care for a safe surgery. If IgM is positive, the patient is probably in the acute phase of infection, and procedure should be the same as that with a patient with positive RT-PCR. If IgM is negative and IgG is positive, the patient has already had contact with the virus and established immunity. However, it is not yet known whether this immunity is temporary or definitive and if the patient could have her surgical procedure performed.
- **Symptomatic patient:** patients with a respiratory symptom or fever should be referred to a hospital area reserved for suspected COVID. In this location, a detailed clinical evaluation and a new classification of the patient's health impairment will be performed by repeating the measurement of temperature, pulse oximetry and respiratory rate, as well as RT-PCR testing. If the test is positive, the decision may be to proceed with the surgery (made in agreement with the patient) and assume greater risks of serious complications in the postoperative period, or to postpone the procedure to a safer time, if possible. If the test is negative, discussing with the patient on the real need for the procedure is interesting, since she is symptomatic. If surgery cannot be postponed, all PPE should be used and conditions for safe surgery adopted.

The recommendation is that anyone working in the operating room wears full PPE, which includes shoe covers, waterproof scrubs, surgical masks, head protection, gloves and eye protection. Limiting the number of people inside the operating room as much as possible and reducing entry and exit movements are also important measures.^10^ ^11^

In patients confirmed with COVID-19 or highly suspicious cases, elective surgery should be postponed until the patient has fully recovered. When surgery cannot be postponed, an exclusive operating room should be used.

Another point to be considered by the medical team when performing gynecological surgical procedures at this time concerns the anesthetic act. It is a known fact that proximity to the patient at this moment for support and the support during the anesthetic block (in case of epidural or epidural anesthesia), as well as manipulation of the upper airways (general anesthesia) can generate and release droplets and aerosols containing the virus in the surgical environment.^12^

## Care in Surgical Management

### Laparoscopic Surgery:

- Use electrosurgical and ultrasound devices in order to minimize the production of plume, with low power setting and avoid long dissection times;
- Whenever available, use a closed smoke evacuation/filtration system with capacity to hold small/ultra-small particles;
- Use laparoscopic suction to remove surgical plume in a closed system before deflating the abdominal cavity; do not vent pneumoperitoneum in the room;
- Use low intra-abdominal pressure (10–12 mmHg), if possible;
- Avoid rapid deflation or loss of pneumoperitoneum, particularly when changing instruments or extracting samples;
- Tissue extraction must be performed with minimal CO2 escape (deflate with a closed smoke evacuation/filtration system or laparoscopic suction before performing minilaparotomy and before vaginal colpotomy);
- Minimize the spray or spread of blood/fluid droplets;
- Minimize the leakage of CO2 from trocars (check seals in reusable trocars or prioritize the use of disposable trocars).

### Laparotomy or Vaginal Surgery

- Perform dissection and bleeding control using non-electrosurgical techniques whenever possible;
- Employ electrosurgical and ultrasonic devices in order to minimize the production of plume, with low power setting and avoid long dissection times;
- Use smoke evacuators with a filter element to absorb small particles whenever possible;
- Use a suction device to remove surgical plume as it is produced;
- Minimize the spray or spread of blood/fluid droplets.

### Hysteroscopy

- The risk of COVID-19 transmission at the time of hysteroscopy with bipolar electrosurgical devices and normal saline as the infusion medium is unknown, but theoretically low;
- Recommendations for the use of PPE follow what has been exposed previously;
- Outpatient surgery should be recommended whenever possible;
- The risks related to laser vaporization and conization procedures are also unknown, and the above recommendations on minimization and suction of surgical plume also apply to these procedures.

## Conclusion

- Surgery for gynecological patients during the COVID-19 pandemic should be evaluated on a case-by-case basis, taking into account patient risk factors and comorbidities and local resources.
- Minimally invasive and vaginal approaches are associated with lower morbidity for the patient in many cases and to shorter length of hospital stay.
- Data on the risk of surgical plume exposure and transmission of COVID-19 are limited.
- There are strategies for all surgical approaches that can help mitigate the risk of exposing teams in the operating room.
- Every patient who will undergo surgery should be tested for SARS-CoV-2;
- If testing is not possible before the surgery, every patient should be considered potentially contaminated and advised on signs and symptoms of SARS-CoV-2 that may appear in the postoperative period;
- Personal Protective Equipment should be offered to the patient and the entire care team for the maximum safety and reduced contagion during all times of hospitalization;
- Every health team should be tested with RT-PCR collection from the nasopharynx or serological tests.
